# Amelioration of Coagulation Disorders and Inflammation by Hydrogen-Rich Solution Reduces Intestinal Ischemia/Reperfusion Injury in Rats through NF-*κ*B/NLRP3 Pathway

**DOI:** 10.1155/2020/4359305

**Published:** 2020-06-10

**Authors:** Ling Yang, Yan Guo, Xin Fan, Ye Chen, Bo Yang, Ke-Xuan Liu, Jun Zhou

**Affiliations:** ^1^Department of Anesthesiology, The Affiliated Hospital of Southwest Medical University, Luzhou, China; ^2^Department of Traditional Chinese Medicine, The Affiliated Hospital of Southwest Medical University, Luzhou, China; ^3^Department of Anesthesiology, Nanfang Hospital, Southern Medical University, Guangzhou, China

## Abstract

Intestinal ischemia/reperfusion (I/R) injury often causes inflammatory responses and coagulation disorders, which is further promoting the deterioration of the disease. Hydrogen has anti-inflammatory, antioxidative, and antiapoptotic properties against various diseases. However, the effect of hydrogen on coagulation dysfunction after intestinal I/R and the underlying mechanism remains unclear. The purpose of this study was to explore whether hydrogen-rich solution (HRS) could attenuate coagulation disorders and inflammation to improve intestinal injury and poor survival following intestinal I/R. The rat model of intestinal I/R injury was established by clamping the superior mesenteric artery for 90 min and reperfusion for 2 h. HRS (10 or 20 mL/kg) or 20 mL/kg 0.9% normal saline was intravenously injected at 10 min before reperfusion, respectively. The samples were harvested at 2 h after reperfusion for further analyses. Moreover, the survival rate was observed for 24 h. The results showed that HRS improved the survival rate and alleviated serum diamine oxidase activities, intestinal injury, edema, and apoptosis. Interestingly, HRS markedly improved intestinal I/R-mediated coagulation disorders as evidenced by abnormal conventional indicators of coagulation and thromboelastography. Additionally, HRS attenuated inflammatory responses and the elevated tissue factor (TF) and inhibited nuclear factor kappa beta (NF-*κ*B) and nucleotide binding and oligomerization domain-like receptor family pyrin domain-containing 3 (NLRP3) inflammasome activation in peripheral blood mononuclear cells. Moreover, inflammatory factors and myeloperoxidase were closely associated with TF level. This study thus emphasized upon the amelioration of coagulation disorders and inflammation by HRS as a mechanism to improve intestinal I/R-induced intestinal injury and poor survival, which might be partially related to inhibition of NF-*κ*B/NLRP3 pathway.

## 1. Introduction

Intestinal ischemia/reperfusion (I/R) injury is a pathophysiological process related to various clinical acute and severe diseases, such as mesenteric arterial thrombosis, hemorrhagic shock, and severe burns. It has the characteristics of high morbidity and high mortality [1–3]. Intestinal I/R injury potentially leads to a compromised mucosal barrier and increases intestinal permeability and translocation of intestinal bacteria. It also can release a great number of inflammatory mediators and cytokines into the blood, causing multiple organ failure [3–7].

Inflammatory cytokines are the major mediators involved in coagulation activation. When the inflammatory response is out of control, the coagulation function is bound to be affected [8, 9]. The body's normal coagulation function is coordinated and maintained by the coagulation system, anticoagulation system, and fibrinolytic system. The coagulation disorder could occur when these three regulatory systems are out of balance. It exists in various diseases involving inflammatory reactions, such as sepsis, I/R, severe burns, and traumatic shock. The process often manifests as bleeding tendency, thrombosis, hemorrhagic-thrombotic syndrome, and so on. Intestinal I/R could irritate the body with infectious factors, which could also cause abnormal blood coagulation [10, 11].

Hydrogen (H_2_) is the oldest and simplest molecule in our Universe. It can protect a variety of organ injuries as well due to its antioxidation, anti-inflammatory, and antiapoptotic effects [12, 13]. Interestingly, H_2_ has a remarkable therapeutic effect on various diseases, including organ ischemia-reperfusion injury and sepsis [14–17]. Previous studies reported that hydrogen-rich solution (HRS) might have a protective effect on intestinal I/R injury, while its molecular mechanism has still remained elusive [18, 19]. More importantly, to date, the effects of HRS on coagulation dysfunction after intestinal I/R injury and the underlying mechanism remained obscure.

Hence, the purpose of this study was to explore whether hydrogen-rich solution (HRS) could attenuate coagulation disorders and inflammation to improve intestinal injury and poor survival following intestinal I/R.

## 2. Materials and Methods

### 2.1. Animals and Experimental Protocol

Adult healthy male Sprague Dawley (SD) rats (body weight, 220-250 g) were provided by the Experimental Animal Center of the Southwest Medical University (Luzhou, China). The animals were maintained under the environmental conditions of (23 ± 2) °C, 12 h : 12 h light-dark cycle, with normal diet, and free access to drinking water. The rats were acclimated for 1 week in the environment before the experiments. The experimental protocols and animal care were approved by the Animal Ethics Committee of the Affiliated Hospital of Southwest Medical University (No. 20180306042). In addition, animal care and handling were performed in accordance with the National Institutes of Health guidelines.

One hundred and fifty-six rats were randomly divided into four groups (*n* = 39 per group): sham-operated group (SHAM), I/R group (I/R), I/R plus hydrogen-rich saline (10 mL/kg, HRS1); and I/R plus hydrogen-rich saline (20 mL/kg, HRS2). In each group, 15 rats were selected and were observed until 24 h after intestinal I/R for survival analysis; 8 rats were chosen, and the samples (blood and intestine) were harvested for morphological evaluation, intestinal edema, and coagulation-related indicators at 2 h after I/R. In addition, due to the limited blood volume of the rats, another 8 rats in each group had to be selected to obtain blood samples (7 mL) for subsequent analysis (inflammation and TF). Finally, blood samples (6 mL) were collected from the 8 remaining rats in each group for thromboelastography and platelet count.

### 2.2. I/R Model and HRS Treatment

Intestinal I/R injury model was established as described previously [4, 6]. Briefly, the rats were fasted for 12 h, with free access to drinking water before surgery. The rats were anesthetized by intraperitoneal injection of 1% sodium pentobarbital (30 mg/kg), and the abdomen was opened along the midline of the abdomen to separate the superior mesenteric artery (SMA). Except for the SHAM group, a noninvasive microarterial clamp was used to clip the SMA for 90 min and reperfusion for 2 h in the other groups. In the SHAM group, only the SMA was isolated, and no clamping was performed. Moreover, rats in the HRS1 group and HRS2 group were injected with HRS (concentration: ≥0.6 mmol/L, ≥0.6 ppm) 10 or 20 mL/kg (Hydrovita Biotechnology Co, Beijing, China) at 10 min before reperfusion through the tail vein, respectively [19, 20]. The rats in the SHAM group and I/R group were injected with 20 mL/kg 0.9% normal saline. The incision was infiltrated with 0.125% ropivacaine to alleviate postoperative pain.

### 2.3. Collection of Specimens

The rats were sacrificed at 2 h after reperfusion with an overdose of sodium pentobarbital (100 mg/kg, intraperitoneal), and blood was taken through the abdominal aorta, 2 cm of small intestine tissue was taken 5 cm from the end of the ileum. The part of the intestinal segment (1 cm) was fixed with 4% paraformaldehyde for morphological analysis; another 1 cm of intestine tissue was for measuring intestinal edema.

### 2.4. Small Bowel Morphology

The paraffin-embedded tissues were stained with hematoxylin-eosin (H&E). Two pathologists, who were blinded to the study groups, observed the small intestine tissue under alight microscope (×200, Olympus, Tokyo, Japan). For each slice, 5 fields were randomly selected, and Chiu's scoring method was used to assess the degree of intestinal injury [21].

### 2.5. Assessment of Edema in Intestine

The severity of intestinal edema was evaluated by the wet/dry (*W*/*D*) weight ratio method [22]. In brief, intestinal segments were harvested, and the surfaces of them were wiped, then the wet weight (*W*) was measured. Furthermore, the tissues were placed in an 80°C oven to dry for 24 hours, and the dry weight (*D*) was obtained. The *W*/*D* weight ratio was then calculated.

### 2.6. TUNEL Assay of Intestine

The terminal deoxynucleotidyl transferase-mediated dUDP-biotin nick end labeling (TUNEL) method with a commercial assay kit (Roche, Indianapolis, IN, USA) was used to detect the apoptosis in intestine. The slices of intestinal tissue were deparaffinized and treated with TUNEL solution as previously described [6]. The total number of cells and positive cells per visual field were calculated. The apoptotic index = 100 × TUNEL positive cells/total cells%.

### 2.7. Detection of Coagulation-Related Indicators

Blood samples (3 mL) were centrifuged at 3000 rpm for 10 min, and related indicators of coagulation function, such as prothrombin time (PT), activated partial thromboplastin time (APTT), thrombin time (TT), fibrinogen (FIB), fibrinogen degradation product (FDP), D-dimer (D-Di), and prothrombin time-international standardization ratio (PT-INR), were detected by an automatic coagulometer (Stago, Paris, France).

### 2.8. Thromboelastography and Platelet Count

Briefly, thromboelastography (TEG) was performed with TEG 5000 Hemostasis System (Hemoscope Corporation, Niles, IL, USA) by adding 20 *μ*L of 0.2 M calcium chloride and 340 *μ*L of citrated whole blood to kaolin. The reaction time (*R*), clotting time (*K*), coagulation angle (*α*-Angle), and maximal amplitude (MA) were recorded [23]. An additional 3 mL of blood was tested for platelet count by an automatic hematology analyzer, BC-6800, Mindray (Shenzhen, China).

### 2.9. Detection of Serum Inflammatory Markers, DAO, and Tissue Factor

A total of 7 mL blood was taken through the abdominal aorta, of which 4.5 mL blood samples were maintained at room temperature for 2 h, centrifuged at 1000 rpm for 20 min, and the supernatant was used to detect the levels of tissue factor (TF); tumor necrosis factor-*α* (TNF-*α*); interleukin-1*β* (IL-1*β*), interleukin-6 (IL-6), and interleukin-10 (IL-10); and the activities of diamine oxidase (DAO) and myeloperoxidase (MPO) in serum. The enzyme-linked immunosorbent assay (ELISA) kits were utilized according to the manufacturer's instructions [(TF, TNF-*α* and IL-6; Cloud-Clone Corp®, Wuhan, China); (IL-1*β* and IL-10; R&D Systems, Inc, Minneapolis, MN, USA); (DAO and MPO; Nanjing Jiancheng Bioengineering Institute, China)]. The results were expressed as pg/mL and U/mL, respectively.

### 2.10. Western Blotting Analysis

The remaining 2.5 mL blood samples were processed according to the animal mononuclear cell separation method, and peripheral blood mononuclear cell layers were extracted. Then, the mononuclear cell layer was added with cell fission extraction protein, and the protein concentration was determined by bicinchoninic acid (BCA) assay. For each group of samples, 40 *μ*g of protein was loaded, electrophoresed on a 10% sodium dodecyl sulfate-polyacrylamide gel electrophoresis (SDS-PAGE) gel, and then, the protein was transferred onto a polyvinylidene fluoride (PVDF) membrane (Amersham Biosciences, NJ, USA) and blocked with 5% milk for 2 h at room temperature. Subsequently, the membrane was incubated overnight at 4°C with primary antibodies forphospho-NF-*κ*B p65 (1 : 200; Abcam, USA), NF-*κ*B p65 (1 : 500; Cell Signaling Technology, Inc., Danvers, MA), NLRP3 (1 : 300), Caspase 1 (1 : 500), and *β*-actin (1 : 300) (Santa Cruz Biotechnology, Inc., CA, USA). It was then washed thrice with Tris-buffered saline with Tween 20 (TBST) and incubated with an anti-rabbit IgG (1 : 5000; Beyotime Biotechnology, Shanghai, China) or anti-mouse IgG (1 : 2500; Santa Cruz Biotechnology, Inc., CA, USA) secondary antibody for 1 h at 37°C. Finally, exposure and development were performed with ECL luminescent reagent, visualized with a protein imaging system, and protein bands were analyzed with Image J software (version 1.31; National Institutes of Health, Bethesda, ML, USA).

### 2.11. Survival Rate

The rats in all groups were observed continuously for 24 hours via video (SMART, Barcelona, Spain) to evaluate survival rate. All rats were allowed food and water *ad libitum*.

### 2.12. Statistical Analysis

Data was presented as mean ± standard deviation (SD) and analyzed using the GraphPad Prism 6 program (GraphPad Software Inc., San Diego, Calif., USA). Survival studies were analyzed using the Kaplan-Meier method followed by a log-rank test. The survival rates were expressed as a percentage and tested by the Fisher exact probability method. For all other data, one-way analysis of variance (ANOVA) with the Bonferroni post hoc test was used to compare differences. Correlation between different variables was assessed by Pearson coefficient [17]. *P* < 0.05 was considered to be statistically significant.

## 3. Results

### 3.1. HRS Improved the Survival of Rats after Intestinal I/R

**Figure 1:**
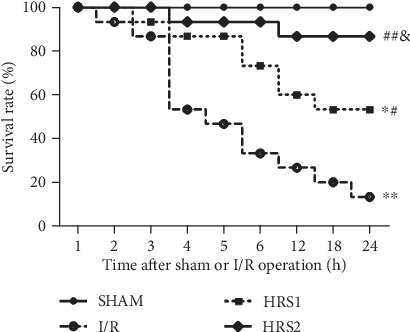
HRS improved the survival of rats after intestinal I/R. Rats subjected to intestinal ischemia for 90 minutes by occluding the superior mesenteric artery (SMA) were received normal saline (vehicle) or HRS (10 or 20 mL/kg of body weight) via the tail vein at 10 min before reperfusion and were observed for 24 hours. Data are presented as the survival percentage of animals. The survival rate is estimated and compared by the Kaplan-Meier method and the log-rank test (*n* = 15/group). ^∗^*P* < 0.05, ^∗∗^*P* < 0.01 vs. SHAM group; ^#^*P* < 0.05, ^##^*P* < 0.01 vs. I/R group; ^&^*P* < 0.05 vs. HRS1 group.

As illustrated in Figure 1, the survival rate at 24 h was significantly reduced in rats subjected to an intestinal I/R event (13.33%, 2 of 15 rats) compared to sham controls (100%, 15 of 15 rats; *P* < 0.01). However, HRS treatment significantly increased the 24-h survival of rats to 53.33% (8 of 15 rats) in the HRS1 group and 86.67% (13 of 15 rats) in the HRS2 group, respectively (*P* < 0.05 or *P* < 0.01). Moreover, 24-h survival of rats in the HRS2 group was higher than that in the HRS1 group (*P* < 0.05). The results showed that HRS could improve the survival of intestinal I/R rats in a dose-dependent manner.

### 3.2. HRS Reduced Small Intestinal Injury, Edema and Serum DAO Activity

**Figure 2:**
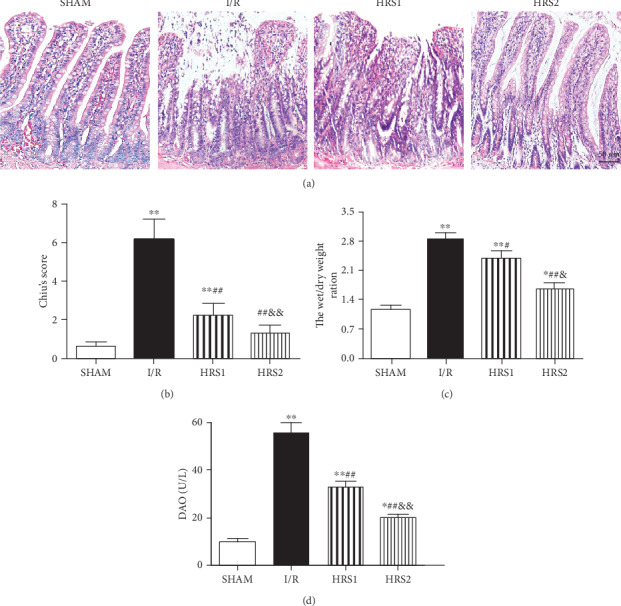
HRS alleviated the histopathological damage in the intestine. (a) Representative images were shown for the pathological changes of intestinal mucosal tissues (hematoxylin and eosin staining, original magnification ×200). (b) The histopathological score of intestine (Chiu's score). (c) The *W*/*D* weight ratio. (d) Serum DAO activities. The data were represented as mean ± SD (*n* = 8/group). ^∗^*P* < 0.05, ^∗∗^*P* < 0.01 vs. SHAM group; ^#^*P* < 0.05, ^##^*P* < 0.01 vs. I/R group; ^&^*P* < 0.05, ^&&^*P* < 0.01 vs. HRS1 group. Scale bar = 50 *μ*m.

As shown in Figure 2, the results of H&E staining showed that the histopathological structure of intestinal mucosa was normal in the SHAM group, the villi and glands were neat, and Chiu's score was significantly lower than that in the I/R group (*P* < 0.01). However, the epithelial cell shedding was observed in small intestinal mucosa of rats in the I/R group, and Chiu's score was higher. Chiu's score in the HRS1 group or HRS2 group was noticeably lower than that in the I/R group (*P* < 0.05 or *P* < 0.01). There was no significant difference between the HRS1 group and HRS2 group (Figures 2(a) and 2(b)). In addition, compared with those of the SHAM group, the *W*/*D* ratio of intestine and serum DAO activities in the I/R, HRS1, and HRS2 groups were all significantly higher (*P* < 0.05 or *P* < 0.01). However, the *W*/*D* ratio and DAO activities in the HRS groups were significantly lower than those in the I/R group. There was significant difference between the HRS1 group and HRS2 group (*P* < 0.05 or *P* < 0.01) (Figures 2(c) and 2(d)).

### 3.3. HRS Attenuated Apoptosis of Intestine

**Figure 3:**
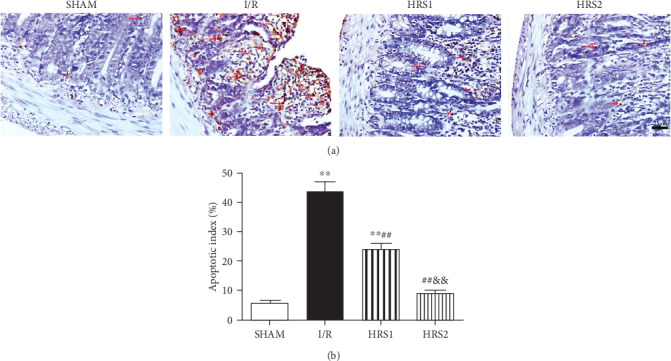
HRS attenuated apoptosis in intestinal tissues. (a) Representative images for intestinal apoptotic cells (original magnification ×400). TUNEL-positive cells with dark brown nuclei indicated apoptosis (red arrows). (b) The quantitative analysis of apoptotic index among different groups. The data were represented as mean ± SD (*n* = 8/group). ^∗∗^*P* < 0.01 vs. SHAM group; ^##^*P* < 0.01 vs. I/R group; ^&&^*P* < 0.01 vs. HRS1 group. Scale bar = 25 *μ*m.

As shown in Figure 3, few TUNEL-positive cells were detected in the SHAM group, the apoptotic index was significantly higher in the I/R group than that in the sham group (*P* < 0.01). Compared with the I/R group, the apoptotic index was significantly lower in the HRS1 and HRS2 groups (*P* < 0.01). The apoptotic index showed no significantly statistical difference in the HRS1 and HRS2 groups (Figure 3).

### 3.4. HRS Improved Coagulation Disorders

**Figure 4:**
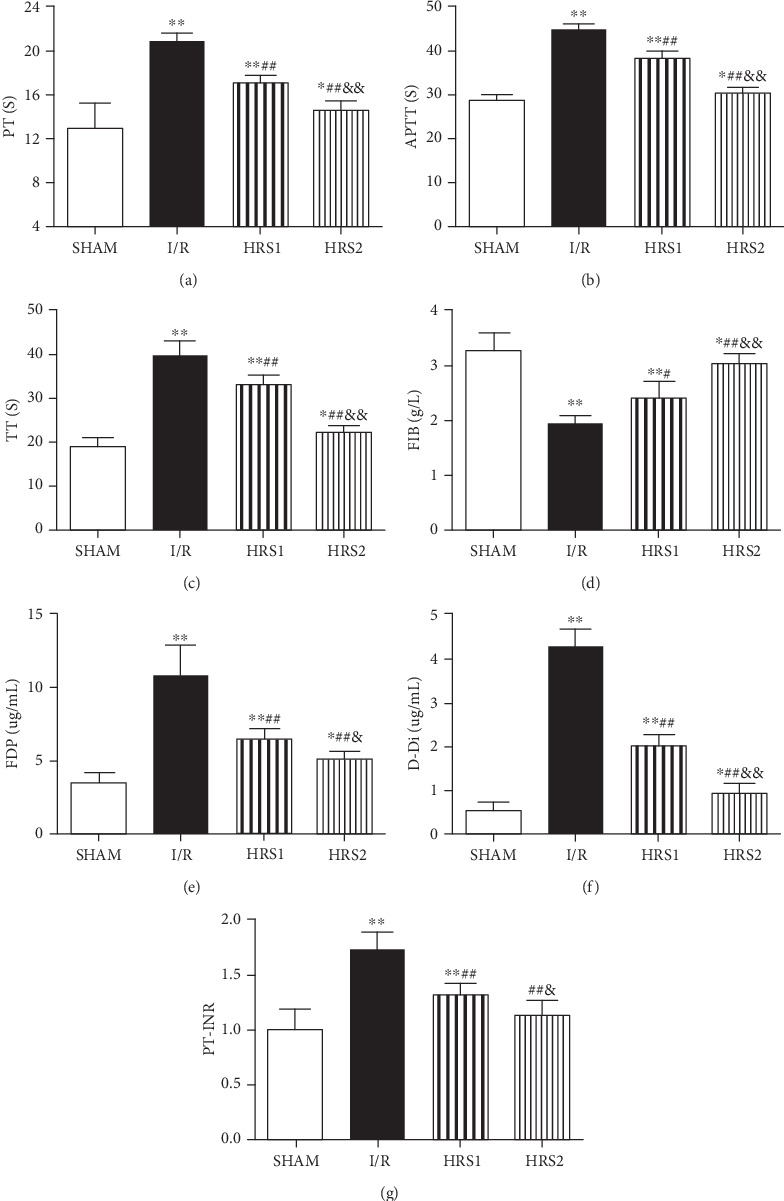
HRS improved coagulation disorders. (a) Prothrombin time (PT), (b) activated partial thrombin time (APTT), (c) thrombin time (TT), (d) fibrinogen (FIB), (e) fibrinogen degradation product (FDP), (f) d-dimer (D-Di), (g) prothrombin time-international standardization ratio (PT-INR). The data were represented as mean ± SD (*n* = 8/group). ^∗^*P* < 0.05, ^∗∗^*P* < 0.01 vs. SHAM group; ^#^*P* < 0.05, ^##^*P* < 0.01 vs. I/R group; ^&^*P* < 0.05, ^&&^*P* < 0.01 vs. HRS1 group.

Compared with the SHAM group, the levels of PT, APTT, TT, FDP, D-Di, and PT-INR in the I/R group were increased, while the FIB level was decreased (*P* < 0.01). After HRS treatment, the levels of PT, APTT, TT, FDP, D-Di, and PT-INR were attenuated, whereas the FIB level was elevated (*P* < 0.05 or *P* < 0.01). The above-mentioned factors also showed significantly statistical difference in the HRS1 and HRS2 groups (*P* < 0.05 or *P* < 0.01) (Figure 4).

### 3.5. Effects of HRS on Thromboelastography and Platelet Count

**Figure 5:**
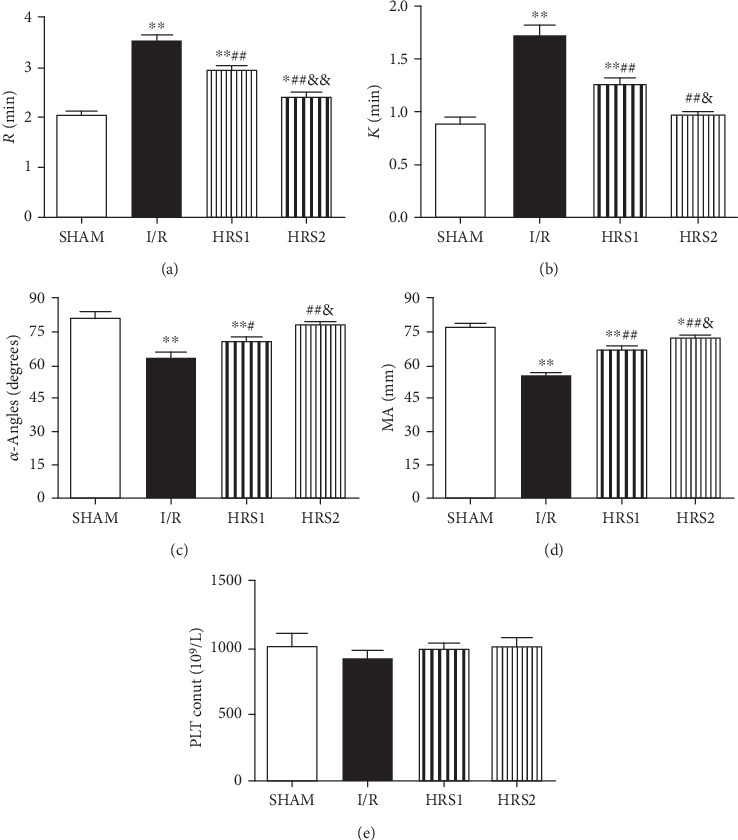
Effects of HRS on thromboelastography (TEG) and platelet count. (a–e) were *R*, *K*, *α*-Angles, MA, and PLT count, respectively. The data were represented as mean ± SD (*n* = 8/group). ^∗^*P* < 0.05, ^∗∗^*P* < 0.01 vs. SHAM group; ^#^*P* < 0.05, ^##^*P* < 0.01 vs. I/R group; ^&^*P* < 0.05, ^&&^*P* < 0.01 vs. HRS1 group.

Herein, *R* and *K* values were increased after intestinal I/R injury, while *α*-angle and MA values were decreased (*P* < 0.01). Compared with I/R group, *R* and *K* values were reduced, whereas *α*-angle and MA values were remarkably increased in the HRS1 and HRS2 groups (*P* < 0.05 or *P* < 0.01). In addition, the above-mentioned factors also showed significantly statistical difference in the HRS1 and HRS2 groups (*P* < 0.05 or *P* < 0.01). However, there was no significant difference in platelet count among all groups (*P* > 0.05) (Figure 5).

### 3.6. Effect of HRS on Serum TNF-*α*, IL-6, IL-1*β*, IL-10, MPO, and TF Levels

**Figure 6:**
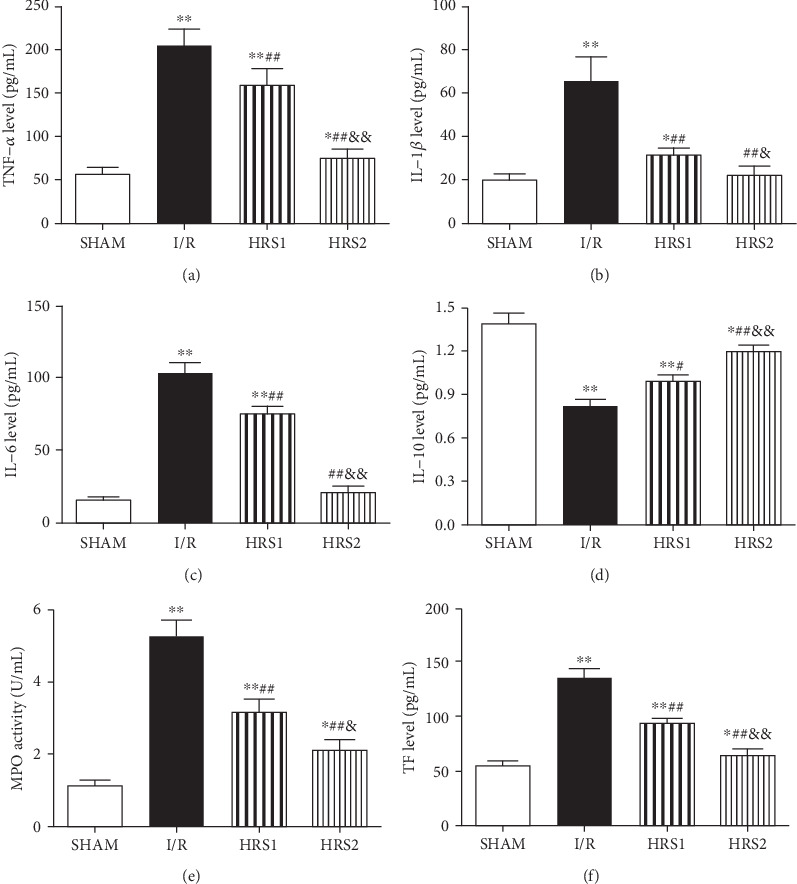
HRS reduced inflammatory stress and TF level. (a–f) were TNF-*α*, IL-1*β*, IL-6, IL-10, MPO, and TF levels, respectively. The data were represented as mean ± SD (*n* = 8/group). ^∗^*P* < 0.05, ^∗∗^*P* < 0.01 vs. SHAM group; ^#^*P* < 0.05, ^##^*P* < 0.01 vs. I/R group; ^&^*P* < 0.05, ^&&^*P* < 0.01 vs. HRS1 group.

Compared with the SHAM group, the levels of TNF-*α*, IL-6, IL-1*β*, MPO, and TF in the serum of rats in the I/R group were elevated, while the levels of IL-10 were reduced (*P* < 0.01). HRS treatment caused significant decrease in TNF-*α*, IL-6, IL-1*β*, MPO, and TF and significant increase in IL-10 when compared to the I/R group (*P* < 0.05 or *P* < 0.01). The improvements of the above-mentioned factors were more obvious in the HRS2 group than those in the HRS1 group (*P* < 0.05 or *P* < 0.01) (Figure 6).

### 3.7. Correlation Analysis

**Figure 7:**
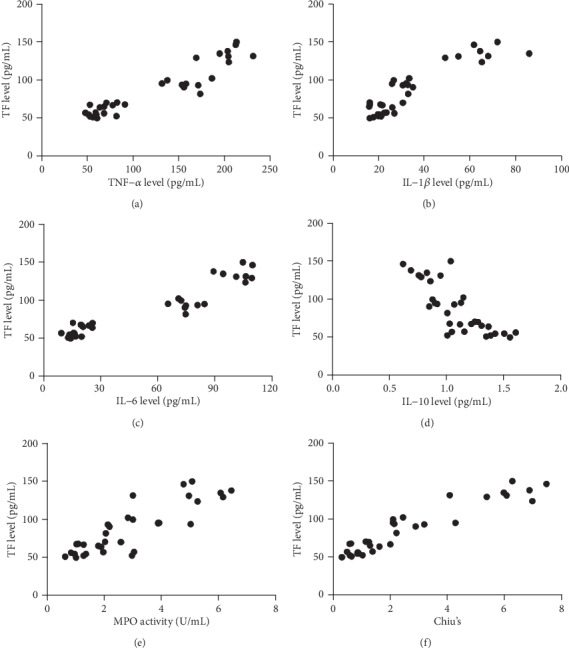
Correlation analysis. Correlations (*N* = 32) between TF level and TNF-*α* level (a), TF level and IL-1*β* level (b), TF level and IL-6 level (c), TF level and IL-10 level (d), TF level and MPO activity (e), TF level and Chiu's score (f).

To determine the relationship between tissue factor and inflammation or intestinal injury, we further analyzed the correlation between them. As illustrated in Figure 7, overall, TF was positively correlated with TNF-*α* (*r* = 0.9007, *P* < 0.01), IL-1*β* (*r* = 0.8467, *P* < 0.01), IL-6 (*r* = 0.8974, *P* < 0.01), MPO (*r* = 0.8552, *P* < 0.01), and Chiu's score (*r* = 0.9399, *P* < 0.01), while TF was negatively correlated with and IL-10 (*r* = −0.7915, *P* < 0.01).

### 3.8. HRS Inhibited the NF-*κ*B and NLRP3 Pathway

**Figure 8:**
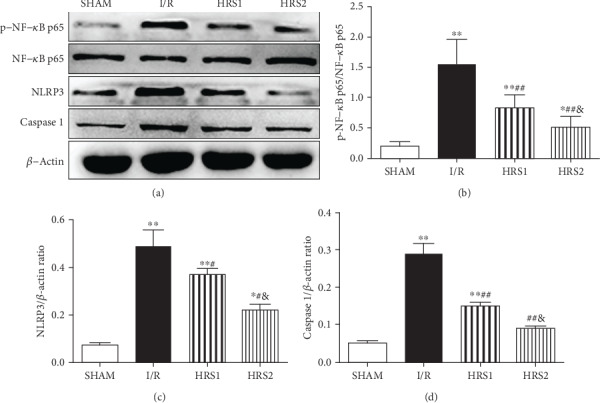
HRS inhibited NF-*κ*B and NLRP3 inflammasome activation. Western blot was used to detect the expression levels of phospho-NF-*κ*B p65, NF-*κ*B p65, NLRP3, and Caspase 1 proteins in PBMCs (a–d). The data were represented as mean ± SD (*n* = 8/group). ^∗^*P* < 0.05, ^∗∗^*P* < 0.01 vs. SHAM group; ^#^*P* < 0.05, ^##^*P* < 0.01 vs. I/R group; ^&^*P* < 0.05 vs. HRS1 group.

NF-*κ*B and NLRP3 signaling pathways were involved in the inflammatory response to acute intestinal injury. To investigate the possible mechanism of HRS in improving intestinal I/R injury and coagulation function, we further studied the expressions of phospho-NF-*κ*B p65 (p-NF-*κ*B p65), NF-*κ*B p65, NLRP3, and Caspase 1 in peripheral blood mononuclear cells. It was disclosed that intestinal I/R injury could up-regulate the expressions of p-NF-*κ*B p65, NLRP3, and Caspase 1 as compared to the SHAM group (*P* < 0.01), while HRS could significantly down-regulate the corresponding expressions in a dose-dependent manner as compared to the I/R group (*P* < 0.05 or *P* < 0.01) (Figure 8).

## 4. Discussion

Intestinal I/R injury is associated with increases in luminal epithelial permeability and ingress of bacterial molecules (e.g., enterotoxin) or bacteria themselves which can result in systemic inflammatory responses, multiple organ failure, and even leading to death [1–3]. In the present study, Chiu's score, the *W*/*D* ratio of intestine, and serum DAO activities increased, indicating that intestinal I/R injury caused a great damage to the intestine, and the model was successfully established [4, 6, 22]. The present results also demonstrated that HRS could attenuate intestinal injury, as evidenced by the improved morphological changes and survival rate. Interestingly, an important finding was that HRS might significantly improve intestinal I/R-mediated coagulation disorders and inflammation, at least in part, by inhibition of activated NF-*κ*B/NLRP3 pathway.

Intestinal I/R injury may cause not only damage to organs and tissues, but also coagulation disorders. The coagulation function of it is regulated by the coagulation, anticoagulation, and fibrinolytic systems, which would affect each other and maintain a dynamic balance under physiological conditions [24]. If any of the three is abnormal and the balance is broken, bleeding or thrombosis may occur. PT and APTT reflect the activity of coagulation factors in the exogenous and endogenous coagulation pathways, respectively. If the coagulation factors are consumed, they may be significantly prolonged. FIB and TT are related to the final stage of coagulation, that is, fibrinogen becomes fibrin. FDP is a degradation product of fibrin or fibrinogen; D-dimer is an important landmark substance for forming thrombus and dissolving fibrin. It can act as a marker for early diagnosis of acute mesenteric ischemia [25]. PT-INR is mainly used to evaluate the anticoagulant level of anticoagulant drugs (e.g., Warfarin). If it exceeds the upper limit, there is a risk of bleeding, and if it is lower than the lower limit, the anticoagulation may be insufficient [26]. The principle of thromboelastography is to detect and quantify dynamic changes of the viscoelastic properties of a blood sample during clotting under low shear stress [23]. During the interaction between inflammation and coagulation, inflammatory mediators may facilitate thrombus formation through at least three pathways at the same time, including up-regulation of procoagulant pathways, down-regulation of physiological anticoagulant mechanisms (antithrombin, protein C system, and tissue factor pathway inhibitor), and inhibition of fibrinolysis [8, 9].

The present study confirmed that after intestinal I/R injury, PT, APTT, and TT were prolonged and FDP and D-Di levels could be increased; besides, PT-INR was elevated and FIB level was decreased. *R* and *K* values were additionally increased according to thromboelastography, while the *α*-angle and MA values were decreased. The above-mentioned findings suggested that the coagulation disorders occurred. The results were consistent with previous studies [10, 11]. Additionally, after intestinal I/R injury, the levels of TNF-*α*, IL-1*β*, IL-6, and MPO in blood were noticeably increased, while the level of IL-10 was decreased dramatically.

TNF-*α* and IL-6 are classic proinflammatory factors in the inflammatory phase. MPO, as an inflammatory biomarker, can reflect leukocyte infiltration. The monocyte-macrophage system may synthesize and release TNF-*α*. When TF expression is up-regulated, it can activate proteinase-activated receptor 1 (PAR-1) on the surface of monocytes. TNF-*α* may activate the cytokine cascade and induce monocytes to express TF, thereby forming a vicious cycle of inflammatory cascade and activation of the coagulation system [27]. Under stimulation of inflammatory factors, the expression of TF is significantly increased in endothelial cells and monocytes, activating the coagulation system through the exogenous coagulation pathway [28, 29]. Under mediation of calcium ions, activated coagulation factor FX and activated coagulation factor FV can form a complex and may generate a small amount of thrombin and simultaneously activate endogenous coagulation pathway, which can mediate the coagulation cascade and coagulation dysfunction [26]. In the activation of inflammatory response, TF assumes the task of connecting inflammation and coagulation. Inflammatory factors, such as TNF-*α*, IL-1*β*, and IL-6, can stimulate monocytes to up-regulate TF express. A recent report has shown that TF is regarded to be the primary initiator of coagulation in severe infection. Local activation of coagulation can be prevented by inhibiting TF level in models of sepsis [30]. Interestingly, in our study, TF level was positively correlated with Chiu's score, which indicated that TF level could also be elevated by intestinal I/R. Previous study showed that the mentioned process was closely associated with the expression of TF in peripheral blood mononuclear cells [27, 29, 30]. TF level was also positively correlated with TNF-*α*, IL-1*β*, IL-6, and MPO levels and was negatively correlated with IL-10, indicating that the TF level was closely associated with the inflammatory factors, which may cause coagulation disorders.

NF-*κ*B is one of the key pathways connecting inflammation and immunity, especially the modulation of proinflammatory factors [31]. Under pathological conditions, such as inflammation, hypoxia, and infection, NF-*κ*B will break away from the binding of its inhibitory protein and enter the nucleus, resulting in a series of effects, such as participating in transcription of the TF gene [28]. Activation of the NF-*κ*B pathway can be mediated by toll-like receptors (TLRs), interleukin 1 receptor (IL-1R), tumor necrosis factor receptor (TNFR), and antigen receptors. Typical stimulating signal molecules are TNF-*α*, IL-6, IL-1*β*, lipopolysaccharide (LPS), and bacterial cell wall [32]. In the rat model of intestinal I/R injury, the expression levels of p-NF-*κ*B p65, NLRP3, Caspase-1, and inflammatory factors were increased. It is hinted that NF-*κ*B/NLRP3 pathway might be involved in intestinal I/R injury. The NLRP3 inflammasome was also shown to mediate hepatic injury induced by intestinal I/R [4]. Therefore, it can be concluded that NF-*κ*B and NLRP3 inflammasome may be a therapeutic target for disease management [33, 34]. If it could inhibit the activation of the NF-*κ*B/NLRP3 pathway, it could not only attenuate the inflammatory response and protect the intestine but also inhibit the TF level and weaken coagulation disorders.

Molecular hydrogen was previously taken as an inert gas into account, while a research reported that it has antioxidant, anti-inflammatory, and antiapoptotic properties, with organ protection for a variety of acute and chronic diseases [12, 13]. In an animal model of intestinal I/R injury, H_2_ can simultaneously reduce local intestinal damage and distant organs damage [12, 18, 19]. In addition, H_2_ can inhibit the activation of NF-*κ*B and NLRP3 pathways, which has a certain therapeutic effect on other diseases. The present experiment revealed that Chiu's score after HRS treatment was reduced, and the coagulation function was improved. The PT, APTT, TT, FDP, D-Di, and PT-INR were significantly lower than those in the intestinal I/R group. FIB level, *α*-angle, and MA values were increased, while *R* and *K* values were decreased. Additionally, HRS significantly reversed the above-mentioned trend of inflammatory factors induced by intestinal I/R.

Platelets are often activated by trauma, infection, etc. and participate in anti-infection, hemostasis, and repair processes. The main mechanisms of thrombocytopenia in septic rats include bone marrow suppression and neonatal thrombocytopenia, immune-mediated platelet destruction, and platelet depletion [35]. However, in the rat model of intestinal I/R injury, there were no statistically significant differences in platelet count among all groups, which was consistent with a previous study [36]. We speculated that the cause might be (at least in part) inflammatory exudation after intestinal I/R, which leads to blood concentration, abnormal distribution of platelets in the body, platelet aggregation, and increased attachment. A recent study demonstrated that hydrogen can inhibit platelet activation and reduce thrombus formation in a thrombosis model [37]. Coagulation disorders can be associated with several factors, including coagulation factors and platelets. In the present study, when platelet count was close to normal level, coagulation dysfunction occurred in rats, suggesting that multiple factors might be involved in coagulation dysfunction during intestinal I/R injury. Hydrogen improved coagulation dysfunction, which might be related to inhibiting inflammatory response. However, the degree of platelet activation during intestinal I/R injury, the level of consumption, and changes in its function still require to be further studied.

There could be some limitations in the current study. Firstly, regarding therapeutic effects of HRS in improving intestinal I/R-induced coagulation disorders, only the inflammatory response was observed. Therefore, further investigation is needed to explore the other functions of HRS as well as its role in other inflammatory conditions. Secondly, this study focused on the short-term effect of HRS on intestinal I/R-induced coagulation disorders and poor survival, while the long-term impact of HRS is yet to explore. The survival rate might be changed in prolonged time in the I/R and HRS treatment groups, and the optimal therapeutic windows of HRS for improving coagulation disorders and survival rate are required further exploration. Finally, two different dosages of HRS were determined based on previous studies [17, 38], without exploring possible effects of HRS at additional dosage on coagulation disorders induced by intestinal I/R injury. The optimal dosage of HRS needs further investigation in future studies.

## 5. Conclusion

In summary, the present study confirmed that intestinal I/R could cause coagulation disorders and the activation of NF-*κ*B and NLRP3 inflammasome in rats. Interestingly, HRS could strikingly improve intestinal I/R-induced intestinal injury and poor survival, which might be partially related to ameliorating coagulation disorders and inflammation via inactivation of NF-*κ*B/NLRP3 Pathway. That is an important finding, indicating that HRS might have clinical potentials for critically ill patients associated with intestinal I/R to improve coagulation disorders and mortality.

## Data Availability

The data used to support the findings of this study are included within the article.
